# Key predictors of food security and nutrition in Africa: a spatio-temporal model-based study

**DOI:** 10.1186/s12889-024-18368-2

**Published:** 2024-03-22

**Authors:** Adusei Bofa, Temesgen Zewotir

**Affiliations:** 1 School of Mathematics, Statistics and Computer Science, University of KwaZulu-Natal, Westville campus, Durban, South Africa; 2 School of Mathematics, Statistics, and Computer Science, University of KwaZulu Natal, Westville campus, Oliver Tambo Building, Durban, South Africa

**Keywords:** Spatio-temporal model, Bayesian Poisson model, Principal component analysis (PCA), Spatial conditional autoregressive, Food Security and Nutrition, Africa

## Abstract

There is voluminous literature on Food Security in Africa. This study explicitly considers the spatio-temporal factors in addition to the usual FAO-based metrics in modeling and understanding the dynamics of food security and nutrition across the African continent. To better understand the complex trajectory and burden of food insecurity and nutrition in Africa, it is crucial to consider space-time factors when modeling and interpreting food security. The spatio-temporal anova model was found to be superior(employing statistical criteria) to the other three models from the spatio-temporal interaction domain models. The results of the study suggest that dietary supply adequacy, food stability, and consumption status are positively associated with severe food security, while average food supply and environmental factors have negative effects on Food Security and Nutrition. The findings also indicate that severe food insecurity and malnutrition are spatially and temporally correlated across the African continent. Spatio-temporal modeling and spatial mapping are essential components of a comprehensive practice to reduce the burden of severe food insecurity. likewise, any planning and intervention to improve the average food supply and environment to promote sustainable development should be regional instead of one size fit all.

## Introduction

The incorporation of spatio-temporal factors in modeling severe food insecurity and nutrition across Africa captures the complexity and heterogeneity of food insecurity dynamics across the continent. This allows for more accurate identification of the dynamics of severe food insecurity and the factors that contribute to it, which can help inform more targeted and effective interventions.

Despite the continent being home to some of the world’s most fertile lands, a significant portion of its population still faces food insecurity. Accordingly, ensuring food security remains a fundamental challenge for many African nations. This situation is marked by the limited availability of sufficient, high-quality, and nourishing food that can adequately fulfill the dietary requirements for a healthy and active lifestyle [1]. The factors contributing to food insecurity in Africa are many and complex, but poverty, climate change, conflict, poor infrastructure, and inadequate investment in agriculture are among the most significant ones [2].

Addressing Africa’s food insecurity requires action at the local, regional, and global levels. Such action may include investing in infrastructure, agriculture, and food production and promoting artisanal producers while expanding access to food for those who need it most. To this end, the Comprehensive Africa Agriculture Development Programme (CAADP) was introduced by the African Union with the aim of promoting agricultural investment and enhancing food security throughout the continent [3]. Food security is also closely linked to other Sustainable Development Goals, including eradicating poverty (SDG 1), achieving gender equality (SDG 5), and promoting health and well-being (SDG 3) [4].

While some African nations have made progress in addressing food security challenges, many others still face significant obstacles. To ensure food security for all, continued efforts and investment will be necessary to address the underlying issues. Nicholson, Stephens [5] reviewed 91 studies and 26 papers on household food security models and found that only a few works used statistical models to identify the components of food security. Instead, most publications focused on access, availability, and usage indicators, with the majority of research being centered on individual households rather than on regional food security. Many studies have also focused on evaluating food security and nutrition (FSN) in Africa, including work by Li and Zhang [6], Waha, Van Wijk [7], Wegenast and Beck [8], and Yuan, NourEldeen [9].

Cooper, Brown [10] reviewed factors that are linked to food security for 16,152 abstracts from 3297 publications published between 1975 and 2018. They indicated links between conflict, the economic downturn, climate change, and food security concerning Africa. Instead of taking into account spatio-temporal impacts, the bulk of the publications under study exclusively evaluated spatial effects. Once more, in a review on food security and nutrition in Africa, none of the investigations were done with the spatiotemporal impacts in mind while defining correlates of food security [5]. Both Nicholson, Stephens [5] and Cooper, Brown [10] justified broadening the range of metrics utilized as explanatory factors when studying food security and nutrition.

Previous publications on food security and nutrition (FSN) indicators in Africa, such as those by Cooper, Brown [10] and Nicholson, Stephens [5], have used criteria that do not provide a comprehensive view of FSN in the continent. The selection of variables has often been influenced by technical considerations, such as the issue of multicollinearity, which limits the range of indicators that can accurately reflect the underlying sources of variation in FSN in Africa. Therefore, it remains crucial to identify significant elements of FSN to understand the shared dynamics of the continent and create effective coping mechanisms.

The exclusion of pertinent spatiotemporal elements, as indicated by Kassouri and Okunlola [11] could result in inconsistent and ineffective estimations. It is important to identify food security correlates from the Bayesian perspective because, as Calderazzo, Wiesenfarth [12] point out, the frequentist approach used in previous works [5, 10] has some limitations (Lack of subjectivity, difficulty in dealing with prior information, and non-robustness), and the challenges in creating efficient, dependable computing techniques for the ICAR(intrinsic conditional autoregressive) models, especially in the frequentist framework [13].

Despite the availability of metrics provided by the Food and Agricultural Organization of the United Nations (FAO), there is still a lack of in-depth analysis of food security and nutrition (FSN) in Africa as a whole. Questions such as changes in Africa’s food security since the beginning of the twenty-first century, the variables that affect these changes, and the distribution(geographic variation or similarity) of relative risk across the continent remain unanswered. To better understand the complex trajectory and burden of FSN in Africa, it is crucial to consider space-time factors when modeling and interpreting food security. Therefore, our goal is to detect the most important factors that contribute to FSN in Africa, while also taking into account the spatio-temporal variations of food security across the continent. By doing so, we aim to provide a framework to better understand the dynamics of the high prevalence of severe food insecurity observed in many regions of Africa.

## Data and statistical method

To support the monitoring and achievement of Sustainable Development Goal 2(a world without hunger, food insecurity, and undernourishment), FAO is required to make data and information available. Accordingly, FAO has got metadata data on Africa’s food and nutrition security. This study covered the period from 2000 to 2019. To handle missing values, particularly in the context of repeated measures or longitudinal data where missingness is common, we utilized the missForest method. This method is based on the random forest algorithm and has been described in detail in our earlier work [14]. Bofa and Zewotir [15] intensively explored the data and by employing principal component analysis (PCA) they generated components that comprehensively described all the metrics (variables) established by the Food and Agricultural Organization of the United Nations (FAO) for food and nutrition security in Africa. Building upon their exploration and PCA results we examine the spatiotemporal dynamics of food security and nutrition (FSN) in Africa.

A total of 1080 data points were gathered, comprising information from 54 different nations on the continent. This dataset comprised 42 indicators or variables that were particularly pertinent to different facets of food security and nutrition, as defined by the FAO concerning Africa. The FAO utilizes the Food Insecurity Experience Scale (FIES) to measure food insecurity. To ensure consistency and comparability across countries, the FIES Survey Module is administered to nationally representative samples of the adult population. The results at the national level are then adjusted to a global reference scale [16]. According to FAO, ECA [17], severe food insecurity is notably prevalent in Africa. Therefore, a valuable metric for assessing food security and nutrition at the regional level is the number of individuals classified as severely food-insecure. The response metric used is the number of severely food-insecure individuals. Severe food insecurity occurs when people are at serious risk of running out of food, experiencing hunger, and, in the most extreme cases, going days without eating [17].

To identify the essential factors related to food security and nutrition, Allee, Lynd [18] emphasized the utility of employing a convergence of evidence strategy involving multiple metrics. In this context, the original set of 40 variables from the FAO dataset was subjected to Principal Components Analysis (PCA) [14]. This approach was adopted to mitigate information loss and address issues related to multicollinearity within the data. Ultimately, ten factors were selected as explanatory variables: nutrient intake, average food supply, consumption status, childcare, caloric losses, environment, undernourishment, food or nutritional stability, adequate dietary supply, and newborn feeding practices. These factors collectively accounted for approximately 74.6% of the total variance in the dataset [14].

The principal component analysis revealed that the first component (PC1) is strongly correlated with indicators such as undernourishment, wasting in children under 5, stunting in children under 5, and obesity in adults. PC1 represents nutrient intake and explains 19.26% of the total variation. The second principal component (PC2) is associated with factors like food supply, food production, and dietary energy and protein supply. It explains 14.96% of the variation and measures the average food supply in Africa. PC3 captures the consumption status of Africans, including factors like GDP per capita, stunting and overweight in children, and obesity prevalence in adults. PC3 explains 10.96% of the variation. PC4, explaining 5.71% of the variance, relates to childcare factors. PC5 (5.51%) is linked to caloric losses, PC6 (4.47%) to the environment, PC7 (3.72%) to undernourishment, PC8 (3.43%) to food stability, PC9 (3.39%) to dietary supply adequacy, and PC10 (3.19%) to feeding practices among infants. These components are correlated with specific factors that collectively capture the complexity of food security and nutrition in Africa as defined by FAO.

The modeling formulation is based on the spatiotemporal framework, which combines a spatial conditional autoregressive (CAR) prior and an autoregressive (AR) process. This method considers both the spatial and temporal interconnections present in the data. The CAR prior is used to represent the spatial component of the data, capturing the spatial autocorrelation between adjacent locations. Meanwhile, the AR process is utilized to model the temporal component, describing the temporal autocorrelation through time [19]. This method can produce more precise and accurate estimates of regression coefficients, particularly when dealing with severe food security counts over time, by incorporating the spatial and temporal correlation between neighboring locations and time periods.

Several comparable models have been used in earlier studies, including Knorr-Held and Besag [20], and Pandey and Tolani [21]. While separable models have been utilized in certain studies (e.g. [19, 22]). , their application to address severe food security and nutrition issues in Africa is rare.

In Bayesian statistics, when constructing a prior distribution for spatially autocorrelated random variables, a common approach is to combine a uniform prior that spans from negative to positive infinity for the intercept (mean) with the intrinsic conditional autoregressive (ICAR) distribution. Leroux, Lei [23] proposed a remarkable modification to conditional autoregressive (CAR) models that we incorporated into our work. Their suggestion involves using the Eq. 

1 $$\text{Q}\left(\text{W}, \rho \right)= \rho \left({\varvec{W}}_{\varvec{d}} - \mathbf{W}\right)+ \left(1 - \rho \right)\mathbf{I}$$

Where, ${\varvec{W}}_{\varvec{d}}$ represents a diagonal matrix of the weighted average value derived adjacency, and **W** is the n dimensional adjacency matrix. **I** represent the identity matrix of size $N\times N$, and 1 is an $N\times 1$ vector of ones. The parameter *ρ*, which controls the spatial correlation strength, takes on values between 0 and 1, inclusive. This results in a CAR distribution ${\varphi }_{i}|\rho , {\tau }^{2},{\varphi }_{j},j\ne i \sim N\left(\frac{\rho \sum _{j=1}^{n}{w}_{ij}{\varphi }_{j}}{\rho \sum _{j=1}^{n}{w}_{ij}+1-\rho } ,\frac{{\tau }^{2}}{{\uprho }\sum _{j=1}^{n}{w}_{ij}+1-\rho } \right)$. In this context, ${w}_{ij}$ refers to the element in the nth dimensional adjacency matrix **W** corresponding to the *i*th row and *j*th column. The random effects have a spatial variance of ${\tau }^{2}$and autocorrelation of $\rho$

The spatial weight matrix related to the units $i$ and $j$ is represented by each item ${w}_{ij} \in$** W**. The constituent of ${w}_{ij}$ is $(i,j)$, which remains the neighborhood matrix with $54\times 54$ dimension. The matrix’s nonzero entries so reveal whether the two places are neighbors. Typically, the weighted matrix is written as:

$$s_{ij}=\left\{\begin{array}{lc}1&if\;areas\;i\;and\;j\;are\;neighbours\\o&ortherise\end{array}\right.$$

The dataset’s spatial autocorrelation is verified by applying Moran’s I =$\frac{n}{{s}_{0}}\frac{\sum _{ij}({{w}_{ij}(x}_{i}-\mu )({x}_{j}-\mu ))}{{\sum }_{i}{({x}_{i}-\mu )}^{2}}$, ‘n’ represents the count of points under investigation, ${x}_{i}{x}_{j}$ denote the observed values at two distinct points, $\mu$ denote the expected value of ‘$x$ and ${w}_{ij}$ signify the spatial weight element. Moran’s I range [-1,1], large values of the relevant metrics are close to other large value clusters when the value is 1, while large values are close to low values when the value of the Moran’s I is -1 [24, 25].

For this study ${Y}_{it}$ follows a Poisson distribution where ${Y}_{it}$ represent the number of severe food insecurity individuals in a country $i$ $(i=1,2,\dots,54)$ in year $t(t=\text{1,2},\dots , 20)$ and ${n}_{it}$ be the population size at country $i$ during time $t$. The food security and nutrition covariates (components) are represented by the design matrix $\varvec{X}$ and $\varvec{\beta }$ is a vector of the related fixed effects parameters:

2 $${\text{l}\text{o}\text{g}(\mu }_{it}){=\eta }_{it}=\varvec{X}\beta +\text{l}\text{o}\text{g}({n}_{it})$$

Adding the spatio-temporal random effect ${\psi }_{it}$ to Eq. 2, the result is Eq. 3 below

3 $${\text{l}\text{o}\text{g}(\mu }_{it}){=\eta }_{it} {+ \psi }_{it}$$

Due to the multiple aspects of the spatio-temporal random effect ${( \psi }_{it})$, it is crucial to decompose ${ \psi }_{it}$ in order to capture its various spatio-temporal features( spatio-temporal interactions).

### Spatio-tmporal Poisson Linear Trend Model (SPLTM)

Firstly, from Eq. 3

4 $${\psi }_{it}={\omega }_{1}+{\phi }_{i}+({\omega }_{2}+{\vartheta }_{i})\frac{t-\stackrel{-}{t}}{T}$$

Where $\stackrel{-}{t}$ is calculated as $\frac{T+1}{2}$ Here ${\omega }_{1}$ and ${\omega }_{2}$ represent the overall intercept and slope parameters, respectively, and are assigned a flat prior distribution. On the other hand, the incremental trend(slope) and intercept parameters for the ith region, denoted as ${\delta }_{i}$ and ${\vartheta }_{i}$

respectively, are assigned a conditional autoregressive (CAR) prior distribution with different values of *ρ* and ${\tau }^{2}.$ Specifically, the parameters parameters ${\varvec{\phi }=(\phi }_{1},\dots ,{\phi }_{n})$ and ${\varvec{\vartheta }=(\vartheta }_{i},\dots , {\vartheta }_{n})$ follow the CAR prior distributions $\varvec{\phi }\sim CAR\left(\varvec{\phi }\right|{\rho }_{int},{{\tau }^{2}}_{int},\varvec{W})$ and $\varvec{\vartheta }\sim CAR\left(\varvec{\vartheta }\right|{\rho }_{slo},{{\tau }^{2}}_{slo},\varvec{W})$ respectively. Similarly, ${{\tau }^{2}}_{slo},{{and \tau }^{2}}_{int}$ represent the variance parameters. The parameters ${\rho }_{slo}{, \text{a}\text{n}\text{d} \rho }_{int}$ are assigned independent uniform prior distributions in the unit interval (0, 1), indicating that their values can range between 0 and 1. On the other hand, the variance parameters, ${{\tau }^{2}}_{slo},{{and \tau }^{2}}_{int}$ follow the inverse gamma prior distribution. In this model, important trends for severe food insecurity are accounted for and their level of significance is accessed.

### Spatio-temporal Poisson Anova Model ( SPAM)

Knorr-Held [26] proposed a method for analyzing data that incorporates the interaction between space and time by using a model which factors spatial and temporal main effects based on the Analysis of Variance. Equation 5 is derived from Eq. 3 to represent the spatial and temporal effect with attraction.

5 $$\psi_{it}=\varnothing_i+\vartheta_t+\varphi_{it},i=1,\dots,n\,\,\,\,\,\,,\,t=1,\dots,\,\,\,T$$

The three sets of parameters indicated by ${\varnothing }_{i}$, ${\vartheta }_{t}$ and $\varphi_{it}$ in Eq. 5 are all considered as random effects, each having its own unique probability distribution.

$$\begin{array}{c}\varnothing\vert\rho_s,{\tau^2}_s,W\;\sim\;CAR\left(\varphi\vert\rho_s,{\tau^2}_s,\varvec{W}\right)\\\varvec{\vartheta}\vert\rho_T,\tau^2\tau,Z\;\sim\;CAR\left(\varvec{\vartheta}\vert\rho_T,\tau,\varvec{Z}\right)\\\varphi_it\;N\left(0,\tau^2I\right),i=1,\dots,n,\;\;t=1,\dots,T\end{array}$$

Here **Z** is the T ×T matrix that represents temporal adjacency, where each element ${ z}_{ij}$ takes a value of 1 if the absolute difference between *i* and *j* is equal to 1, and 0 otherwise. It is assumed that the interaction effect ${(\varphi }_{it })$ is independent for all values of i and t. The parameters ${\rho }_{s}$ and ${\rho }_{T}$ are assigned uniform priors with a range of values between 0 and 1, similar to before ("Spatio-Tmporal Poisson  Linear Trend Model (SPLTM)" section), while the variance parameters ${{\tau }^{2}}_{s}$, ${{\tau }^{2}}_{T}$, and ${{ \tau }^{2}}_{I}$ are assigned inverse gamma priors.

### Spatio-temporal Poisson Separable Model(STSM)

The separable model, which includes an overall time trend with temporal-specific spatial effects, is an alternative model to the one presented in "Spatio-Temporal Poisson Anova Model ( SPAM)" section. In this case, independent conditional autoregressive (CAR) models are assigned to the spatial effects ${ \varvec{\phi }=(\varphi }_{1t},\dots ,{\varphi }_{nt})$ for each $t=1,\dots ,T$ and to **ϑ**=(${{\upvartheta }}_{1}$,…, ${{\upvartheta }}_{T}$), as shown in the following equation.

6 $$\begin{array}{c}\psi_it=\phi_it+\vartheta_t,i=1,\dots,n,t=1,\dots,T\\\phi_t\vert\rho_s,\tau^2t,W\;\sim\;\mathrm{CAR}\left(\mathrm\phi\vert{\mathrm\rho}_{\mathrm s},\mathrm\tau^2\mathrm t,\;\mathrm W\right),\\\vartheta\vert\rho_T,\tau^2,Z\;CAR(\vartheta\vert \rho_T,\tau^2,Z)\end{array}$$

**Z** refers to the same definition as previously provided in "Spatio-Temporal Poisson Anova Model ( SPAM)" section. The variance parameters ${{\tau }^{2}}_{t}$, where t ranges from 1 to T, and ${\tau }^{2}$, are assigned inverse gamma prior distributions, similar to the previous model. The parameters ${\rho }_{s}$ and ${\rho }_{T}$ are assigned independent uniform prior distributions with a range of values between 0 and 1.

### Poisson temporal model for Spatiotemporal Effect (TMS)

The postulation is made that a particular scenario within the framework of the separable model, as discussed in "Spatio-Temporal Poisson Separable Model (STSM)" section, corresponds to a temporal autoregressive model with a lag of one. In this special case, the parameter ${\vartheta }_{t}$ is equal to zero for all values of t, resulting in the simplification as ${\psi }_{it}={\phi }_{it}$, here ${\vartheta }_{t}=0$ aimed at the entire $t$ and

$${\varvec{\varphi }}_{\varvec{t}}\left|{\varvec{\varphi }}_{t-1}\right., \varvec{W} \sim N\left(\rho T,{\varvec{\varphi }}_{t-1}, {\tau }^{2}\varvec{Q}{\left(\varvec{W},\varvec{\rho }\varvec{s}\right)}^{-1}\right), t=2, \dots ., T$$

$${\varvec{\varphi }}_{1}\left|\varvec{W} \sim N\left(0, {\tau }^{2}\varvec{Q}{\left(\varvec{W},\varvec{\rho }\varvec{s}\right)}^{-1}\right),\right.$$

The precision matrix **Q(W, ρS)** with spatial dependence is defined in Eq. (1), where the temporal autocorrelation is caused by the mean $\rho T,{\varvec{\phi }}_{t-1}$. The prior distributions are assumed to remain unchanged.

### The chain models and implementation

After decomposing the spatio-temporal random effect ${(\psi }_{it})$ and adding it to the linear predictor ${\eta }_{it }$ as shown in Eq. 3, four different models were obtained: **STLTM**, **SPAM**, **STSM**, and **TMS** respectively:

7 $${\text{l}\text{o}\text{g}(\mu}_{it})=\eta_{it}+\omega_1+\phi_i+(\omega_2+\vartheta_i)\frac{t-\overset-t}T$$

8 $$\log\left({\mathrm\mu}_{\mathrm{it}}\right)={\mathrm\eta}_{\mathrm{it}}+\phi_{\mathrm i}+{\mathrm\vartheta}_{\mathrm t}+{\mathrm\varphi}_{{\mathrm{it}}}$$

9 $$\log\left({\mathrm\mu}_{\mathrm{it}}\right)={\mathrm\eta}_{\mathrm{it}}+\phi_{\mathrm{it}}+{\mathrm\vartheta}_{\mathrm t}$$

10 $$\log\left({\mathrm\mu}_{\mathrm{it}}\right)={\mathrm\eta}_{\mathrm{it}}+\phi_{\mathrm{it}}$$

In order to build a Markov chain that converges to the target distribution, Markov Chain Monte Carlo (MCMC) techniques are used. In each iteration, a new sample is suggested from a distribution based on the current state of the chain. The acceptability of the proposed sample is determined by comparing its probability to that of the current state. If the proposed sample has a higher probability, it is accepted; if not, the acceptance is determined by the ratio of the probabilities. Using R statistical software version 4.2.2, we developed an MCMC algorithm that generates 10,000 samples from the posterior distribution with *N* = 120,000, burn.in = 20,000, and thin = 10, and then we quantified the level of uncertainty in the model estimations by computing the 95% credible intervals.

### Model validation

Assessing the model’s performance, especially with new data, is essential. To evaluate the model’s performance using the out-of-sample strategy, this study employed cross-validation, with 70% of the samples used for model training and 30% for testing. Additionally, the study calculated the coverage percentage (CP) for the 95% predictive intervals using the formula $$CP=100\frac{1}{k}{\sum }_{i=1}^{k}I\left({L}_{i}\le {y}_{i}\le {U}_{i}\right)$$, where $${y}_{i}$$ represents the observed value for i=1,…,k and $${(L}_{i},$$$${U}_{i})$$ represents the 100 (1 − α)% predictive interval for predicting $${y}_{i}$$. The indicator function I(·) was also used in the calculation.

## Results

Table 1 presents the estimated parameters of various spatio-temporal interaction models that were fitted to explore the associations between severe food insecurity and different levels of covariates from 2000 to 2019.

**Table 1:** The parameter estimation for spatio-temporal models that explain FSN in Africa with a 95% credible interval

Characteristics	STLTM	SPAM	STSM	TMS
Nutrients Intake	0.044(0.002,0.085)*	0.061(-0.004,0.125)	0.061(0.003,0.177)*	0.042(-0.017,0.100)
Average Food Supplied	-0.238(-0.296,-0.180)*	-0.218(-0.291,-0.144)*	-0.158(-0.220,-0.093)*	-0.225(-0.294,-0.154)*
Consumption Status	0.059(-0.007,0.124)	0.085(0.004,0.156)*	0.116(0.046,0.190)*	0.074(-0.004,0.148)
Child Care	0.048(0.008,0.087)*	-0.014(-0.079,0.050)	-0.016(-0.090,0.056)	-0.005(0.063,0.052)*
Caloric Losses	-0.038(-0.1331,0.023)*	-0.034(-0.137,0.035)	-0.031(-0.128,0.036)	-0.038(-0.139,0.028)
Environment	-0.179(-0.242,-0.115)*	-0.129(-0.209,-0.053)*	-0.121(-0.190,-0.054)*	-0.147(-0.223,-0.070)*
Food Stability	0.128(0.052,0.200)*	0.143(0.046,0.240)*	0.126(0.051,0.199)*	0.136(0.051,0.221)*
Dietary Supply adequacy	0.303(0.343,0365)*	0.255(0.177,0.332)*	0.246(0.180,0.313)*	0.256(0.181,0.329)*
Feeding Practise among Infants	0.048(-0.010,0.105)	0.027(-0.044,0.096)	-0.003(-0.062,0.056)	0.038(-0.031,0.106)
Undernourishment	-0.048(-0.097, 0.000)	-0.657(-0.135,0.004)	-0.119(-0.190,-0.053)	-0.067(-0.130,-0.004)*
$\alpha$	-0.050(-0.260,0.163)			
${{\tau }^{2}}_{intecept}$	1.143(0208,4.416)*			
${{\tau }^{2}}_{slope}$	1.438(0.341,5.388)*			
${\rho }_{intecept}$	0.099			
${\rho }_{slope}$	0.017			
${{\tau }^{2}}_{spatial}$		0.657(0.168,2.038)*		
${{\tau }^{2}}_{Temporal}$		0.144(0.076,0.265)*		
${{\tau }^{2}}_{Interaction }$		0.223(0.166,0.285)*		
${\tau }^{2}$				0.713(0.240,1.993)
${\rho }_{spatial}$		**0.387(0.055,0.891)**	0.023(0.011,0.041)	0.018(0.001,0.067)
${\rho }_{Temporal}$		**0.415(0.037,0.861)**	0.446(0.043,0.872)	0.232(0.010,0.581)
WAIC	2433.588	**2222.700**	3081.113	2230.773

The value of α from the STLT model, which represents the overall trend in FSN across Africa, suggests a slight decline, but this decline is not statistically significant. However, there is substantial variation in trend levels among individual African countries, as indicated by a trend variation level of 1.438, with a credible interval ranging from 0.341 to 5.388. This indicates that there is a weak correlation between trends (0.017). Interestingly, despite this weak correlation, the model evaluating spatio-temporal variation for each year individually has the second highest Watanabe-Akai Deviance Information criteria (WAIC) value among the models (Table 1).

The STSM provides a comprehensive understanding of the yearly changes in FSN across Africa. It captures the spatial and temporal dynamics of FSN, allowing for a detailed analysis of the variations observed. However, it is worth noting that this model has the highest WAIC value among the models evaluated, indicating that it may be more complex and less generalizable than other models.

**Fig. 1:**
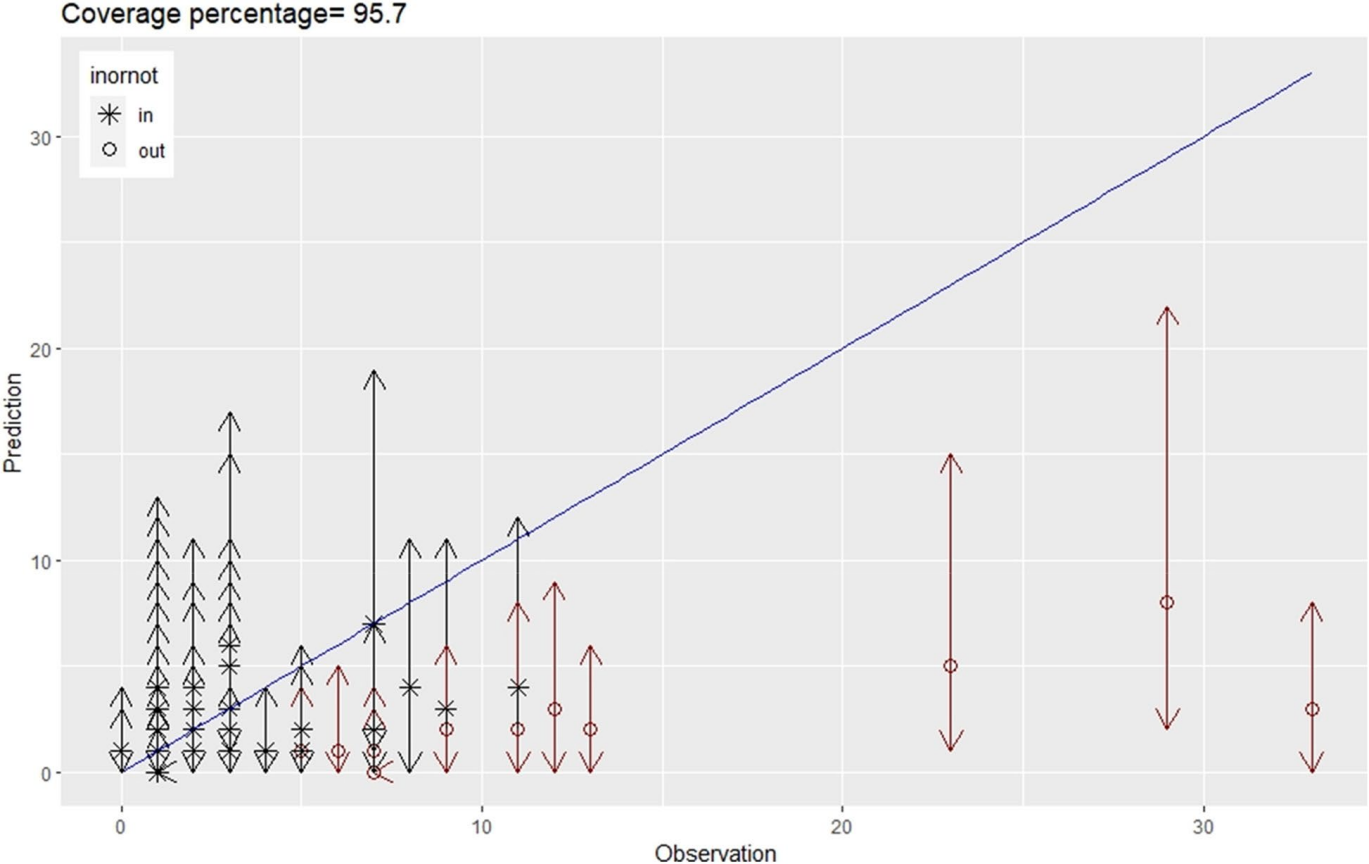
Model validation for the final SPAM model for FSN concerning Africa from 2000–2019

Among the four fitted models, the SPAM Model, which incorporated a spatio-temporal interaction effect, demonstrated the lowest WAIC value of 2222.70. This indicates that the SPAM Model performed better than the other models in identifying the most relevant correlates of severe food insecurity while accounting for the specific spatio-temporal effects in Africa (Table 1). To evaluate the predictive performance of the final SPAM model on new data, a model validation process was conducted, and the results are presented in Fig. 1. The coverage value, which measures the model’s ability to capture the variability in the new data, was computed and found to be close to perfect, with a high coverage percentage of 95.7%. Figure 1 visually supports this finding, providing evidence that the SPAM model effectively predicts severe food insecurity outcomes using new data.

According to Table 1, the average food supply has a statistically significant negative effect on the rate of severe food insecurity, with a coefficient of exp(-0.218) and a 95% credible interval of exp(-0.291, -0.144). This means that for every one-unit increase in the average food supply, there is a decrease of approximately 80.41% in the growth rate of severe food insecurity. Similarly, the environment also has a statistically significant negative effect on the expected rate of severe food insecurity, with a coefficient of exp(-0.129) and a 95% credible interval of exp(-0.209, -0.053).

However, dietary supply adequacy has a positive effect on the rate of severe food insecurity, with a coefficient of exp(0.255) and a 95% credible interval of exp(0.177, 0.332). This means that for every unit increase in dietary supply adequacy, there is a 29.05% increase in the rate of severe food insecurity. Furthermore, the food stability and consumption status also have positive effects on the rate of severe food insecurity, with coefficients of exp(0.143) and exp(0.085) respectively. This suggests that food stability and consumption status are associated with an increase in the rate of severe food insecurity.

Based on the results in Table 1, the spatial correlation parameter $({\rho }_{spatial})$ is estimated to be 0.387 with a 95% credible interval of (0.055, 0.891), indicating a moderate positive spatial correlation. This suggests that neighboring geographical units tend to have similar values for the variables of interest in this study. On the other hand, the temporal autocorrelation $\left({\rho }_{Temporal}\right)$ has a value of 0.415 with a 95% credible interval of (0.077,0.861), which is larger than the spatial correlation after accounting for the spatio-temporal effect. This indicates that there is a positive correlation between observations taken at different time points, which is not explained by the spatial correlation alone. Moreover, our model was able to quantify the spatial variation and temporal variation separately. The spatial variation was estimated to be 0.657, while the temporal variation was estimated to be 0.1440.

**Fig. 2:**
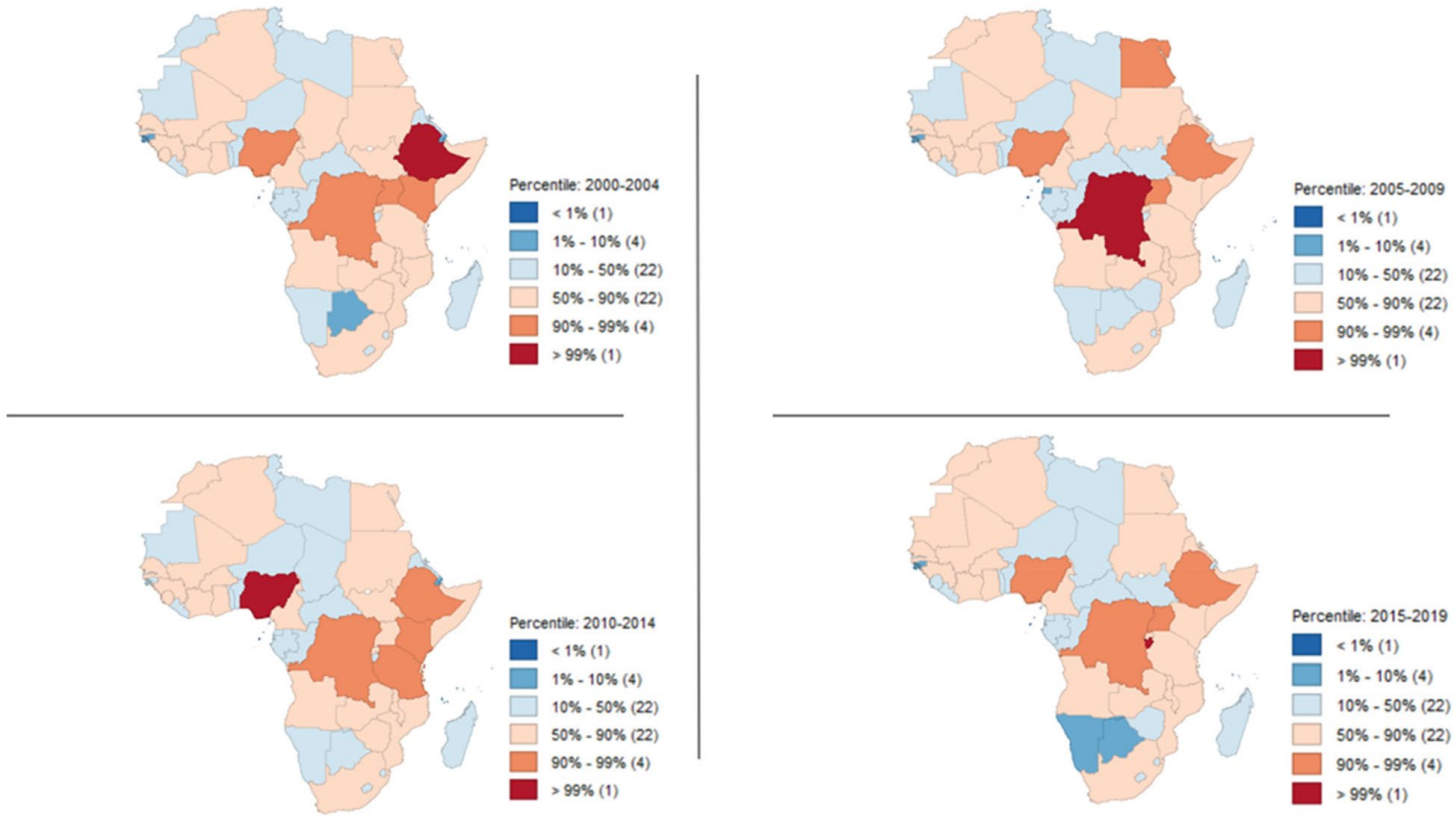
Spatial percentile distribution for aggregated severe food insecurity for the fitted values(SPAM) concerning Africa

Figure 2 provides valuable insights into the spatial and temporal patterns of FSN in Africa, which a visual representation of the spatial and temporal patterns of FSN (food security and nutrition) in Africa is presented. The map shows the percentile values, indicating the relative severity of food insecurity across the continent [27]. The map reveals substantial clustering and continuity in the values over time, suggesting that certain regions consistently experience high or low levels of food insecurity. The map also highlights that high percentiles (ranging from 50 to 99%) are present in approximately half of the countries in Africa (27 out of 54). These high percentiles indicate severe food insecurity and nutrition. According to the percentile distribution, the temporal changes in the number of severe food insecurity cases within each country are influenced by the temporal changes in other countries. This finding is consistent with the results obtained from the SPAM model, which revealed that the spatial correlation and temporal correlation of FSN in Africa are 0.387 and 0.415, respectively, as shown in Table 1.

The trajectory analysis revealed a concerning upward trend, signifying a notable increase in the rate of food insecurity across various regions of Africa. These regions include the western, north-western, central, eastern, and south-eastern parts of the continent. The rise in food insecurity underscores the susceptibility of these regions, emphasizing the immediate necessity for the implementation of effective improvement strategies. It’s worth noting that among the most vulnerable countries in terms of food security are Nigeria, the Democratic Republic of the Congo, Zambia, Uganda, and Ethiopia. This highlights the need for regional strategies such as Trade and Market Integration. This approach will facilitates trade among neighboring countries, reduces trade barriers, and enhances market access. It promotes economic growth and food security by ensuring that surplus production in one region or country can benefit areas with food deficits within Africa. Additionally, the implementation of data-sharing policies among African countries is crucial. Such policies encourage information-sharing mechanisms that improve early warning systems, monitor food prices, and track supply chains. This, in turn, enables swift responses to potential crises and contributes to overall food security in the region. regional rather than unified planning and intervention for the continent need to be implemented.

### Most important spatio-temporal significant predictors in more recent years ( 2018 and 2019)

Once the model was validated, further analysis was conducted to determine if the five identified significant predictors (determinants) of food security remained important in more recent years. Hence, data from 2018 to 2019 was used to assess the stability and generalizability of the model’s findings. This process aimed to assess whether the relationships between these significant predictors and food security held true in recent years, thus validating their ongoing relevance.

**Table 2:** Resent spatio-temporal significant correlates: cross validation (2018 and 2019 data)

Characteristics	Point Estimate	95% Credible Interval
Average food Supplied	1.441	0.533 2.316 *
Consumption Status	0.112	-0.152 0.383
Environment	-0.026	-0.296 0.228
Food Stability	0.399	-0.324 1.126
Dietary Supply Adequacy	0.269	0.099 0.438 *
${{\tau }^{2}}_{spatial}$	0.768	0.165 2.852
${{\tau }^{2}}_{Temporal}$	0.508	0.140 1.676
${{\tau }^{2}}_{Interaction }$	0.350	0.174 0.571
${\rho }_{spatial}$	0.328	0.009 0.900
${\rho }_{Temporal}$	0.425	0.019 0.928

Table 2 displays the outcomes of the analysis conducted on the most spatio-temporal predictors influencing food security in recent years (2018 and 2019). Out of the five variables chosen based on their statistical significance in the SPAM model (as shown in Table 1), the results indicate that Average Food Supplied and Dietary Supply Adequacy remained statistically significant in explaining food security dynamics in recent years. Nevertheless, there is a positive correlation between average food supply and severe food insecurity. This could potentially signify a period of economic downturn or the inefficacy of policies, strategies, and mechanisms regarding average food supply, particularly affecting Africa in recent times. However, Consumption Status, Environment, and Food Stability did not exhibit significance in the context of recent years (2018 and 2019). This suggests that during this particular period, these predictors did not have a substantial impact on food security in Africa, despite their statistical significance in the broader analysis. Furthermore, the analysis revealed a moderate positive spatial correlation of 0.328 and a temporal correlation of 0.425 for the 2018 and 2019 data. The associated variation for these correlations was determined to be 0.768 for spatial correlation and 0.508 for temporal correlation.

## Discussion

We adopted Bayesian Poisson spatio-temporal framework and incorporates fixed effects derived from principal component analysis (PCA). This paper establishes and evaluates 4 different spatio-temporal models (employing statistical criteria) to examine the relationship between severe food insecurity and various covariates which found the SPAM model to perform the best based on WAIC(2222.7), indicating its superior performance (Table 1). The validation results, shown in Fig. 1 for the SPAM model, also support the significance of considering spatio-temporal effects in explaining the variation in severe food insecurity across Africa.According to our model, the significant correlates, even after accounting for spatial variation concerning Africa, include average food supply, consumption status, environment, food stability, and dietary supply adequacy. In this section, these statistically significant variables(PCA Components), directly sourced from the FAO dataset without undergoing PCA transformation, are linked with the core indicators for food security in a broader context within Africa.The study suggests that researchers and stakeholders should take into account these effects to effectively prevent and control severe food insecurity and malnutrition in Africa.

According to our model, severe food security is positively predicted by dietary supply adequacy, food stability, and consumption status. Previous studies such as Deaton and Deaton [28] and Hasegawa, Sakurai [29] have found that an increase in dietary supply adequacy increases the risk of severe food insecurity, while Grote, Fasse [30] found that an increase in food stability and consumption status is associated with an increase in the rate of severe food insecurity which is in line with our finding. These findings suggest that Africa’s average food supply for its population is insufficient, which affects food stability on the continent. This may be due to the impact of climate change on food production and the lack of irrigation facilities in Africa.

The components of dietary supply adequacy are average dietary energy supply adequacy,dietary energy supply used in the estimation of the prevalence of undernourishment, and per capita food supply variability. This result confirms findings from other studies such as those by Bonuedi, Kamasa [31] and Hasegawa, Sakurai [29]. For food stability, the components are rail line density (total route in km per 100 square km of land area), percent of arable land equipped for irrigation, and value of food imports in total merchandise exports. This finding corroborates the finding of previous works [32, 33]. The consumption status identified in our model is linked to gross domestic product per capita, the percentage of children under 5 years of age who are stunted, the percentage of children under 5 years of age who are overweight, the prevalence of obesity in the adult population (18 years and older), and the minimum dietary energy requirement [34, 35]. These variables fall within the FAO domain for food security indicators [14] ,which can have a significant impact on the consumption patterns and nutritional status of individuals in Africa and ultimately affect food security.

Table 1 shows that the average food supply and the environment have a statistically significant negative impact on the rate of severe food insecurity, as indicated by the regression coefficient with a corresponding level of significance.

The components of average food supply, which were found to have a statistically significant negative impact on severe food insecurity, include various factors such as food production value, dietary energy supply, protein supply (both overall and animal-based), cereal import dependency, and fat supply. This finding is consistent with previous studies by Grote, Fasse [30] and Morales, Morales [36]. In addition, the component of the environment was also found to have a significant negative effect on severe food insecurity, with variables such as the percentage of the population using safely managed drinking water and sanitation services being important factors. This is in line with research by Kookana, Drechsel [37] and Nyiwul [38]. Taken together, these findings suggest that allocating resources towards improving average food supply and environmental conditions is crucial for Africa to make progress in reducing the rate of severe food insecurity and malnutrition.

The findings of this study can be used as an essential tool for monitoring and evaluating progress in the fight against severe food insecurity. It also provides a strong basis for designing, modeling, and implementing severe food insecurity control programs.

## Conclusion

In this study, a model-based inference approach was employed to investigate severe food insecurity and malnutrition in Africa, using Bayesian spatio-temporal modeling techniques with fixed effects. The study used Bayesian fixed and random effects (both spatial and temporal) to explore the influence of geographic changes on food insecurity and nutrition in Africa. The selected spatio-temporal model enables simultaneous analysis of pattern persistence over time and the identification of any extraordinary patterns. Furthermore, by incorporating space-time interaction terms, it can potentially identify data clusters. In addition, the study identified and examined significant determinants of food security and nutrition in Africa. The results of the study suggest that dietary supply adequacy, food stability, and consumption status are positively associated with severe food security, while average food supply and environmental factors have negative effects on FSN. The findings also indicate that severe food insecurity and nutrition are spatially and temporally correlated across the African continent. Also, the interaction between space and time is significant. We recommend that the African Union should revamp its Comprehensive Africa Agricultural Development Programme (CAADP) to include components for dietary supply adequacy, food stability, average food supply, and environment to increase agricultural investment and promote food security across the continent.

## Data Availability

The datasets used and/or analysed during the current study available from the corresponding author on reasonable request.
